# Cytokine-induced killer (CIK) cells: from basic research to clinical translation

**DOI:** 10.1186/s40880-015-0002-1

**Published:** 2015-03-05

**Authors:** Yelei Guo, Weidong Han

**Affiliations:** Department of Immunology, Institute of Basic Medicine, Chinese PLA General Hospital, Beijing, 100853 P. R. China

**Keywords:** Cytokine-induced killer cells, Immunotherapy, Antitumor effect, Cancer therapy, Clinical trials

## Abstract

The accumulation of basic researches and clinical studies related to cytokine-induced killer (CIK) cells has confirmed their safety and feasibility in treating malignant diseases. This review summarizes the available published literature related to the biological characteristics and clinical applications of CIK cells in recent years. A number of clinical trials with CIK cells have been implemented during the progressive phases of cancer, presenting potential widespread applications of CIK cells for the future. Furthermore, this review briefly compares clinical applications of CIK cells with those of other adoptive immunotherapeutic cells. However, at present, there are no uniform criteria or large-scale preparations of CIK cells. The overall clinical response is difficult to evaluate because of the use of autologous CIK cells. Based on these observations, several suggestions regarding uniform criteria and universal sources for CIK cell preparations and the use of CIK cells either combined with chemotherapy or alone as a primary strategy are briefly proposed in this review. Large-scale, controlled, grouped, and multi-center clinical trials on CIK cell-based immunotherapy should be conducted under strict supervision. These interventions might help to improve future clinical applications and increase the clinical curative effects of CIK cells for a broad range of malignancies in the future.

## Background

Cytokine-induced killer (CIK) cells are a heterogeneous cell population that was first discovered in the 1990s and can be generated from lymphocytes co-cultured with an anti-CD3 antibody and many other cytokines *in vitro* [1]. Numerous studies have demonstrated that CIK cells exhibit active proliferation and potent antitumor cytotoxicity against multifarious tumor cells *in vitro* and *in vivo* [1,2]. Increasing data show that the antitumor effects of CIK cells rely on a perforin-based mechanism and Fas-Fas ligand interactions [3,4]. CIK cells are also not inhibited by immunosuppressive drugs [5], which makes CIK cells an ideal candidate cell type for cancer therapy.

Theoretically, CIK cell-based adoptive cellular immunotherapy (ACI) could be a curative strategy for cancer. Abundant clinical trials on this therapeutic regimen have been published in the past two decades, confirming its safety and feasibility in cancer patients [6-8]. Several other clinical trials focusing on graft-versus-host disease (GVHD) and viral infections related to this therapy have also been conducted in recent years [9,10].

**Figure 1:**
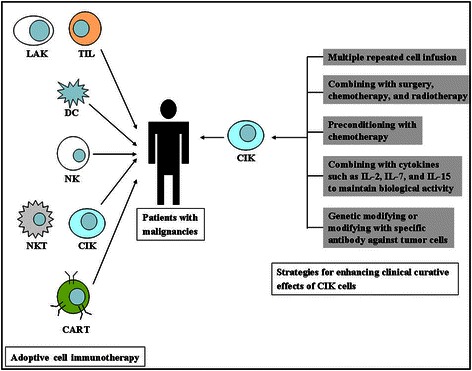
**The present existing adoptive cellular immunotherapy and strategies for enhancing clinical curative effects of cytokine-induced killer (CIK) cells.** CIK cells have become the main adoptive immunotherapeutic cells because of their particular biological characteristics and have been demonstrated to exert their therapeutic function in various malignancies except T-cell lymphoma. Additionally, numerous clinical trials have suggested that some existing regimens using CIK cells can enhance the clinical curative effects on malignant diseases. LAK, lymphocyte-activated killer cells; TIL, tumor-infiltrating lymphocytes; DC, dendritic cells; NK, natural killer cells; NKT, natural killer T cells; CART, chimeric antigen receptor-modified T cells; IL, interleukin.

Given the ongoing investigations of CIK cell-based ACI, this regimen has potentially widespread application prospects in the clinic for most types of cancer. In addition, several strategies to improve the clinical effects of CIK cells have been conducted (Figure 1). For example, CIK cells combined with traditional cancer treatments, including surgery, chemotherapy, and radiotherapy, may achieve the best objective responses in patients [11]. Furthermore, preconditioning chemotherapy, activated cytokines, and specific antibodies could enhance the antitumor ability of CIK cells [12-15]. Recently, attempts at repeated CIK cell infusions have resulted in fewer adverse events and similar clinical curative effects for some malignancies in the clinic compared with genetically modified ACI [16,17]. However, several problems, such as the universal and massive preparation of CIK cells, must be recognized because their resolution could improve the clinical applications of CIK cells and better evaluate overall clinical responses. In addition, the clinical therapeutic procedures of using CIK cells, either combined with chemotherapy or alone as the primary strategy, will be briefly outlined. Taken together, the status quo of CIK cell-based ACI suggests that the use of CIK cells as an effective clinical cancer treatment still has room for improvement. Further large-scale, controlled, grouped, and multi-center CIK cell-based clinical trials are urgently needed.

In this review, we critically summarize current researches on the biological characteristics and recent clinical trials of CIK cells and briefly compare the clinical applications of CIK cells with those of other immunotherapeutic cells. We also present concerns on CIK cell-based ACI drawn from these clinical trials.

## Review

### Biological characteristics of CIK cells

#### Immune phenotype of CIK cells

Up to now, intensive and strict studies on the immune phenotype of CIK cells have been conducted. CIK cells, which are a heterogeneous cell population, comprise CD3^+^CD56^+^, CD3^+^CD56^−^, and CD3^−^CD56^+^ cells [18]. CD3^+^CD56^+^ cells, which are derived from CD3^+^CD56^−^ T cells, are also called natural killer T (NKT) cells and are primarily responsible for non-major histocompatibility complex (MHC)-restricted antitumor activity [19,20]. Furthermore, this subset co-expresses CD2, T-cell receptor (TCR) αβ, and CD8, but not CD16 [21]. In addition, CD3^+^CD56^+^ cells bear the CD27^+^CD28^−^ or CD27^−^CD28^−^ phenotype because they belong to terminally differentiated T-cell populations, whereas CD3^+^CD56^−^ cells are subjected to early differentiation and mainly express the CD27^+^CD28^+^ and CD62L^+^ phenotypes [22,23]. Meanwhile, CD3^+^CD56^−^ cells also express CD4, CD8, and TCRαβ [21,23]. CD3^−^CD56^+^ cells behave similarly to conventional natural killer (NK) cells and express classical NK-cell receptors [23]. In addition to these markers, CIK cells express CD45RA, C-C chemokine receptor (CCR)7, CD11a, macrophage inflammatory protein 1a, perforin, and Fas ligand [19].

#### Cytokine secretion of CIK cells

Numerous studies have indicated that CIK cells express different cytokines, with or without stimulation by tumor cells (Table 1). In the absence of stimulation by tumor cells, CIK cells secrete interferon (IFN)-γ and the highly expressed cytokine receptors interleukin (IL)-2Rβ, IL-2Rγ, IL-10Rα, and IL-10Rβ. However, the expression of cytokines, including IL-2, IFN-γ, tumor necrosis factor (TNF)-α, granulocyte-macrophage colony-stimulating factor (GM-CSF), and IL-4, and cytokine receptors such as IL-2R, IL-4R, IL-12R, and IL-15R, is up-regulated in CIK cells upon stimulation by tumor cells [16,24].

**Table 1:** **Differential cytokine and receptor expression of cytokine-induced killer (CIK) cells upon stimulation by tumor cells**

**Cytokine/receptor**	**Secretion/expression status**	
	**With stimulus**	**Without stimulus**
IL-2	+	+
IFN-γ	+	+
TNF-α	+	+
GM-CSF	+	-
IL-4	+	-
IL-2R	+	+
IL-4R	+	+
IL-10R	-	+
IL-12R	+	-
IL-15R	+	-

IL-2, interleukin 2; IL-4, interleukin 4; IFN-γ, interferon γ; TNF-α, tumor necrosis factor α; GM-CSF, granulocyte-macrophage colony-stimulating factor; IL-2R, interleukin 2 receptor; IL-4R, interleukin 4 receptor; IL-10R, interleukin 10 receptor; IL-12R, interleukin 12 receptor; IL-15R, interleukin 15 receptor. “+” indicates secreted or expressed; “-” indicates not secreted or expressed.

#### Immune function-related gene expression of CIK cells

The immune function-related genes of CIK cells have also been described in recent years. A number of studies have presented that the immune function-related genes of CIK cells are changed under tumor cell stimulation. For example, upon stimulation by acute myelocytic leukemia (AML) cell lines, a CD3^+^CD56^+^ subset of CIK cells highly express various immune function-related genes such as IFN-γ, TNF-α, the cytokine receptor genes IL-7R and IL-12Rβ2, the chemokine and chemokine receptor genes C-C chemokine ligand (CCL)4, CCL5, chemokine (C-X-C motif) receptor (CXCR)3, and CCR1, and the genes encoding granzyme B and caspase-1 [24]. With the exception of these genes, the expression levels of many other genes were up-regulated and those of 7 genes were down-regulated in CIK cells stimulated by AML cells. Furthermore, the genes encoding IFN-γ, GM-CSF, IL-4, IL-8, IL-2Rα, IL-2Rβ, IL-4R, IL-12Rβ, chemokines, chemokine receptors, and TNF were highly expressed in CIK cells under stimulation by acute lymphoblastic leukemia (ALL) cells [22]. Based on these findings, CIK cells may act consistently with T helper 1 and T cytotoxic 1 cell polarization upon stimulation by tumor cells. The expression of related genes in CIK cells seems similar even under stimulation by different tumor cells.

#### Migration and homing potentials of CIK cells

Currently, the migration and homing of CIK cells are being investigated. To date, more and more evidence from animal models has indicated that CIK cells, similar to conventional T cells, can migrate to tumors, tumor-draining lymph nodes, and spleen tissues [25,26]. The distribution and order of flow for CIK cells were related to the immune properties of the cells, the blood supply, and the expression of certain specific chemokines and chemokine receptors for the tumor. The detailed mechanism and efficacy of CIK cell trafficking might involve (1) the up-regulation of specific receptors or ligands on the tumor or tumor-draining lymph nodes to promote the homing, adhesion, and infiltration of CIK cells [25,26] and (2) the chemokines, chemokine receptors, selectins, and adhesion molecules expressed on CIK cells that are involved in the migration of CIK cells across the endothelium [23,25,26]. On the contrary, CIK cells infiltrate normal target tissues to a much smaller extent or transiently for a longer time [25]. This result was confirmed by another animal study. CIK cells are effective against Fas ligand-positive malignant cells and cells with multidrug resistance. It has been observed that a population of CIK cells migrated to tumor sites by 72 h after infusion and remained detectable at these sites for an additional 9 days. Meanwhile, transplanted tumor cells in mice could be significantly destroyed by these CIK cells [27].

#### Cytotoxic activities of CIK cells

Thus far, numerous studies have demonstrated that CIK cells have potent cytotoxic activities against various tumors such as human leukemia, ovarian cancer, lung cancer, liver cancer, cervical cancer, and colorectal cancer; among CIK cells, CD3^+^CD56^+^ cells have the most potent MHC-unrestricted cytolytic effect [19,28]. The antitumor mechanisms of CIK cells include (1) effector cell-target cell contacts through binding of the surface adhesion molecule leukocyte function-associated antigen-1 (LFA-1) on CIK cells to LFA-1 ligands expressed on most susceptible tumor cells, thereby leading to cytotoxicity against tumor cells [3,29]; (2) the cell signaling pathway involving the binding and activity of NK-cell receptors to their ligands, which are highly expressed on tumor cells, resulting in CIK cell activation that leads to degranulation and cytotoxicity against tumor cells [30]; and (3) the induction of tumor cell apoptosis by Fas ligand via the Fas signaling pathway [19]. In addition, applications in an animal model suggested that *in vivo* antitumor effects against various hematopoietic and solid tumors occurred after the infusion of CIK cells [19,27]. A preclinical study reported that the survival of severe combined immunodeficient mice injected with human lymphoma cells could be significantly prolonged after receiving CIK cell infusion [19].

### Clinical applications of CIK cells in disease therapy

The therapeutic potential of CIK cells as a potential adjuvant therapy for cancer has already been well recognized. A number of studies have indicated that therapeutic regimens with CIK cells could be safe and feasible for cancer patients [6,7,31]. The antitumor effects of CIK cells have been observed on both hematologic malignancies and many solid tumors [6,7,16,17]. In addition, the anti-virus and anti-GVHD potentials of CIK cells could be extensively used for clinical applications [9,10].

At present, the clinical effect of CIK cells has been supported by an increasing number of clinical trials on various types of tumors. A total number of 40 registered clinical trials involving different hematologic malignancies and solid tumors can be found on the website ClinicalTrials.gov (http://www.clinicaltrials.gov) (Table 2). More importantly, CIK cells can be successfully generated from patients with various malignancies, regardless of whether they have received chemotherapy or other treatments. Below, we rigorously summarize recent clinical trials with CIK cells for various disorders.

**Table 2:** **Current status of clinical trials of CIK cells**

**Target disease**	**Registered trial**	**Phase**	**Combined treatment**	**Enrolled patients (cases)**
Lung cancer	NCT01498055	II/III	None	120
	NCT01631357	II/III	Chemotherapy	200
Small cell lung cancer	NCT01592422	II	Best supportive care	60
	NCT01481259	II/III	Chemotherapy	120
Non-small cell lung cancer	NCT01902875	Undefined	Chemotherapy	100
	NCT01871480	II	Gefitinib	50
Hepatocellular carcinoma	NCT01821482	II	DC	100
	NCT01758679	IV	Chemotherapy	120
	NCT01749865	III	None	200
	NCT00769106	III	None	200
Renal cell carcinoma	NCT01924156	I/II	Adenovirus-transfected autologous DC	30
	NCT00862303	I/II	DC + cytokines	100
	NCT01240005	I/II	None	30
Nasopharyngeal carcinoma	NCT01655628	II	Chemotherapy	40
	NCT01821495	II	DC	100
Esophageal carcinoma	NCT01691625	Undefined	Chemoradiotherapy + DC	50
	NCT01691664	Undefined	Radiotherapy + DC	40
Colorectal cancer	NCT01839539	II	DC	60
	NCT02202928	II	Chemoradiotherapy + DC	60
	NCT01929499	II	None	210
Gastric cancer	NCT02215837	II	DC	40
	NCT01783951	I/II	Chemotherapy + DC	70
Pancreatic cancer	NCT01781520	I/II	Chemotherapy + DC	30
Cholangiocarcinoma	NCT01868490	I/II	None	13
High-risk soft tissue sarcoma	NCT01898663	I/II	Adenovirus-transfected autologous DC	30
Malignant glioma	NCT01235845	I/II	DC	30
Triple negative breast neoplasms	NCT01395056	Undefined	Chemotherapy + DC	50
Solid tumor	NCT01914263	I	None	40
Solid tumor and B-cell lymphoma	NCT01799083	I/II	Chemotherapy	100
Hematologic malignancies	NCT00460694	I/II	None	20
	NCT00477035	I/II	Chemotherapy	22
	NCT01186809	II	None	39
	NCT00186342	Undefined	Ablative allo-HCT	120
Multiple myeloma	NCT00185757	I	None	20
Acute leukemia	NCT01956630	I/II	Genetically modified DC	25
Chronic myeloid leukemia	NCT00815321	II	None	11
Acute myeloid leukemia and myelodysplastic syndrome	NCT00394381	I/II	None	17
Myelodysplastic syndrome	NCT01392989	II	Chemotherapy	21
Lymphoma	NCT01828008	Undefined	None	20
Malignant tumor	NCT01884168	Undefined	DC	30

These data were searched on 15 July 2014 from the website ClinicalTrials.gov (http://www.clinicaltrials.gov). The key word “cytokine-induced killer cells” was used. DC, dendritic cells; HCT, hematopoietic cell transplantation.

#### Safety in patients with malignancies after receiving CIK cell infusion

Safety is the greatest advantage of the adoptive infusion of CIK cells in patients with malignant diseases. In recent years, an increasing number of animal studies have indicated that a regimen involving the adoptive infusion of CIK cells confers considerable antitumor effect without severe adverse events in animals with malignancies [25,32,33]. For example, no infusion-related acute and delayed toxicities developed after human CIK cell infusion in nude mice bearing the human colorectal cancer cell line SW1116 [32]. In a murine lymphoma-inoculated H-2^d^ recipient mouse model transplanted with hematopoietic stem cells from H-2^b^ donor mice, no or minimal GVHD developed after CIK cells expanded from donors were infused [33]. Moreover, Nishimura *et al*. [25] infused lethally irradiated BALB/c mice with allogeneic CIK cells generated from FVB mice. After receiving increasing doses of CIK cells, these mice showed minimal signs of GVHD and mild weight loss, and all mice survived.

On the basis of animal studies, a large number of clinical trials have indicated that adverse events seldom occurred after the infusion of CIK cells; most of the adverse events were minor, including mild fever, chills, fatigue, and GVHD. In a phase I clinical trial, 3 out of 10 patients developed fevers that spontaneously resolved after receiving autologous CIK cells electroporated with the *IL-2* gene [8]. The fever was likely due to the transfected *IL-2* gene. Very recently, the same group summarized adverse events in 206 patients with various hematologic malignancies enrolled in 17 clinical trials [34]. After receiving autologous or allogeneic CIK cells, minimal adverse events, such as mild fever (37.5°C-40°C), transient ventricular arrhythmias, chills, fatigue, and GVHD, developed and were easily treated with symptomatic regimens [34]. Chung *et al*. [35] reported that only grade 3 toxicities, including general weakness in 2 patients and thrombocytopenia in 1 patient, occurred after treating 20 advanced pancreatic cancer patients with autologous CIK cells. In another phase I study, autologous CIK cells were given to 10 patients with renal cell carcinoma after radical nephrectomy [36]. Adverse events included mild arthralgia, laryngeal edema, fatigue, and low-grade fever and developed in only 3 patients during the course of CIK cell infusion; these events were either resolved without intervention or treated with symptomatic treatments [36].

#### Feasibility of treating malignant diseases with CIK cells

More excitingly, over the last few decades, many clinical trials have indicated that the adoptive infusion of CIK cells is feasible for patients with malignancies. The first clinical trial using CIK cells modified with the *IL-2* gene for the treatment of 10 patients with metastatic renal carcinoma, colorectal cancer, and lymphoma reported that 1 patient acquired a complete remission [8]. In addition, the same group summarized the overall benefits in 206 patients with various hematologic malignancies who had recently received autologous or allogeneic CIK cells and were enrolled in 17 clinical trials [34]. More than 40% of the patients attained overall disease control (complete remission in 58 patients, partial remission in 15 patients, and stable disease in 11 patients) after the infusion of CIK cells. In our group, we used autologous CIK cells to treat 2 patients with recurrent or refractory AML [16]. After 4 infusions within 4 months, the leukemia burden in the peripheral blood of 1 patient was dramatically decreased. Aside from hematologic malignancies, the use of CIK cells has also been explored in clinical trials as a treatment for various solid tumors. In one report, 79 patients with stage III non-small cell lung cancer who were treated with dendritic cell-CIK cell infusions and chemotherapy had a longer median disease-free survival and a lower 3-year cumulative recurrence rate than the patients who were treated with chemotherapy alone (28 months vs. 22 months; 47.37% vs. 76.92%) [37]. In another study, 156 patients with gastric cancer after operations were divided into two groups: 75 received CIK cell infusions plus chemotherapy, and 81 underwent chemotherapy alone [7]. The 2- and 5- year survival rates were higher in the combination treatment group than in the chemotherapy group (73.5% vs. 52.6%; 40.4% vs. 23.9%). Based on the above-mentioned data, CIK cell-based ACI is a highly feasible regimen for tumor therapy in the clinic. Especially in combination with other treatments, the clinical curative effect of CIK cell-based ACI becomes more prominent.

#### CIK cells in viral infections

Current studies have indicated that hepatitis B virus (HBV) replication in hepatocellular carcinoma (HCC) patients can be suppressed by CIK cells [10]. HBV-induced chronic hepatitis B is a major threat to human health worldwide, and HBV infection also increases the risk of developing HCC [38]. However, less dramatic treatments have been rather effective in recent years. Still, various evidence indicates that the CD3^+^CD56^+^ subset of CIK cells, which are referred to as NKT cells and play an important role in anti-viral infections, might be the main driver of the anti-HBV infection effect [19,20,39,40]. In brief, these issues make it important to obtain definitive evidence of the efficacy of CIK cells for treating viral infections.

#### CIK cells in GVHD

Allogeneic transplantation is a common treatment for hematopoietic malignancies, and GVHD, a life-threatening symptom of allogeneic transplantation, is a main cause of transplantation-related death [41,42]. Because of the extensive research and application of CIK cell therapy for hemotologic diseases, we separately listed and discussed the relationship between CIK cells and GVHD. Moreover, traditional immunosuppressants frequently fail to suppress GVHD; thus, a safe and effective approach is needed to better treat patients with GVHD. Recent studies have demonstrated that NKT cells can suppress transplant rejection [39,40]. Therefore, the anti-GVHD properties of CIK cells are similar to those of NKT cells because of CD3^+^CD56^+^ CIK cells, here referred to as NKT cells. Although hematopoietic stem cell transplantation (HSCT) is an effective treatment for hematologic malignancies, allogeneic immune activation might promote GVHD [43,44]. With the transplantation of CIK cells after HSCT, the risk of GVHD decreases [9]. Based on the information above, CIK cell therapy is regarded as an effective immunosuppressant of GVHD, which often occurs following allogeneic transplantation.

### Comparison of clinical applications of CIK cells and other adoptive immunotherapeutic cells

Over the last few decades, the idea of ACI for cancer has been slowly becoming a reality. Adoptive immunotherapeutic cells have mainly included tumor-infiltrating lymphocytes (TIL), lymphokine-activated killer (LAK) cells, CIK cells, and chimeric antigen receptor-modified T (CART) cells. Studies demonstrated that ACI with TIL cells for melanoma patients achieved more exciting clinical benefits based on the *in vitro* selection of tumor-specific T cell lines and MHC-restricted antitumor function [45,46]. Nevertheless, the extremely low number of TIL cells or tumor cells that lost the expression of a specific antigen or MHC molecule render TIL cell-based ACI procedures not suitable for cancer patients [26]. LAK cells were used for the first prototype of ACI; however, LAK cells expanded insufficiently *ex vivo*. The low cytolytic effects of LAK cells *in vivo* hampered their clinical applications as a cancer therapy and have led to few successful clinical trials [47-49]. Based on LAK cells, CIK cells were generated and shown to have a substantial increase in cytotoxic effect and proliferative response, which has recently been widely applied to treat tumors in the clinic [1]. Compared with traditional immunotherapeutic cells, CART cells can specifically target tumor cells and persistently proliferate, survive, and display antitumor abilities [50,51]. The one-time infusion of these cells may decrease the infusion frequencies and cell numbers, further making CART cells an ideal and novel candidate cell type for cancer therapy. To summarize the above findings, CIK cell-based ACI is a cancer treatment that can be used in some cancers with unclear tumor target genes, and repeated infusions of CIK cells or CIK cell infusion in combination with other therapeutic regimens may achieve unexpected clinical effects. Moreover, CART cell-based ACI is more suitable for cancers with clear tumor target genes; this treatment could possibly achieve a cure.

Importantly, successful ACI is based on a detailed estimation of a particular disease, including tumor evaluation, tumor incidence, tumor growth characteristics, and a portfolio of alternative treatment strategies [52]. In accordance with the biological characteristics of these immunotherapeutic cells, CIK cells are more suitable for tumors with no specific tumor antigen, small lesions, slow proliferation, early disease stages, and alternative treatments. In recent years, a number of clinical studies have indicated that repeated CIK cell infusions could be well tolerated and produce good clinical outcomes for patients with hematologic malignancies such as AML and diffuse large B-cell lymphoma [16,17]. This treatment is able to overcome the defect that a single infusion cannot offer a sufficient or effective number of cells to eradicate tumor cells. This is because CIK cells belong to a subpopulation of terminally differentiated effector T cells that have restricted survival *in vivo* [53]. In addition, using CIK cell-based ACI to consolidate therapy is very advantageous for patients to obtain overall disease control as a clinical outcome with no or minimal adverse events.

### Potential concerns of CIK cells in the clinic

#### Uniform criterion and large scale of CIK cell preparations

At present, CIK cells can be easily generated from various sources, mainly including autologous peripheral blood and bone marrow [54]. The infusion of these cells is well tolerated and does not cause severe adverse events, such as fever, myalgia, flu-like symptoms, and fatigue. Further, this strategy can be easily applied in advanced and/or metastatic malignant diseases [55,56]. Many clinical trials with CIK cell-based ACI have demonstrated potentially widespread future applications. Nevertheless, the prognosis and clinical curative effects are difficult to assess because CIK cells were prepared from autologous sources among different cancer patients. In addition, the adoptive infusion of allogeneic CIK cells could cause GVHD despite their weak alloreactivity and low GVHD potential *in vivo*. Therefore, searching for universal sources for CIK cell preparation may be a better approach to resolving these problems.

Compared with unrelated donor peripheral blood and bone marrow, umbilical cord blood (UCB) has several advantages, including easy acquisition, rapid availability, and low to no risk of adverse events such as GVHD [57]. More recently, some studies have indicated that CIK cells prepared from UCB are suitable for immunotherapy against both malignant and nonmalignant disorders [11,58]. UCB-derived CIK (UCB-CIK) cells have similar biological characteristics to traditional CIK cells obtained from peripheral blood [11]. Moreover, some clinical studies have demonstrated that treating malignancies with UCB-CIK cells shows promising efficacy with negligible adverse events, such as transient fever, most of which disappeared without pharmacologic intervention [11,58]. Because of this finding, this treatment strategy with UCB-CIK cells may represent a safe and feasible approach to treat patients with various malignancies. Furthermore, some studies have indicated that universal T-cell products could be generated from allogeneic donors based on the knockdown of the human leukocyte antigen genes to avoid NK cell-mediated recognition and lysis [59,60]. In addition, hematopoietic stem cells and other precursor cells have been confirmed as potentially being among the starting cell types of T-cell products [61-63]. Accordingly, treatment with CIK cells generated from these sources may also be ideal and safe for cancer patients because of the lower alloreactivity of these cells. In conclusion, the infusion of allogeneic CIK cells generated from the sources mentioned above might not trigger alloreactivity *in vivo*, although these cells were prepared from an unrelated donor. The prognosis and clinical curative effects could be easily evaluated under that CIK cells were generated from universal sources.

However, there are many studies reporting that CIK cells can be generated under different culturing procedures, such as activation and proliferation with IFN-γ, anti-CD3 antibody, and IL-2, as well as additional activated cytokines [1,50]. However, the similar antitumor ability, immune phenotypes, cytokine secretions, and antitumor patterns of these CIK cells may be different. Therefore, the preparation of universal CIK cell products under uniform culturing criteria would likely enhance the uniformity of the biological characteristics of the cells to allow for better evaluation of the clinical curative effects. Using streamlined and centralized manufacturing of CIK cells, including large-scale preparations and cryopreservation, these cells could probably be commercialized anywhere, thereby increasing convenience and flexibility for cancer patients.

#### Cancer therapy with CIK cells combined with chemotherapy or alone as a primary strategy

For advanced malignant diseases, the efficacy of chemotherapy is often low because of the resistance of tumor cells against antitumor drugs. Therefore, new antitumor approaches to overcome and/or ameliorate this resistance to chemotherapeutic drugs in cancer patients are urgently needed. Recently, studies have indicated that immunotherapy with CIK cells may promisingly overcome chemotherapy resistance by stimulating the immune system and enhancing its antitumor abilities [64,65]. Nevertheless, CIK cell-based ACI is often regarded simply as a complementary treatment for cancer therapy by certain clinical researchers and patients and may only be used to treat cancer patients if their state is otherwise incurable. Excitingly, abundant clinical researches have indicated that CIK cell-based ACI has potential beneficial clinical effects for patients with refractory and recurrent malignancies. Because of those findings, combining the therapeutic regimens of CIK cells with chemotherapy or using them alone as a primary strategy may result in better clinical benefits for cancer patients. In the future, several tasks should be urgently addressed, including the following aims: (1) to establish the benefits of using CIK cells as a primary strategy, combined with chemotherapeutic drugs or alone; (2) to observe the clinical curative effects and adverse events in the processing of these regimens; and (3) to analyze and compare these effects with the clinical benefits of traditional chemotherapy.

## Conclusions

Considering the preclinical and clinical studies together, CIK cell-based ACI has demonstrated considerable benefits in various malignancies. The significant outputs do appear promising for clinical curative effects. However, many of the problems mentioned above must be resolved to acquire a better clinical curative effect of cancer treatment using CIK cells. Therefore, it is of particular importance to implement large-scale, controlled, grouped, and multi-center clinical trials to enhance the therapeutic effects of CIK cell-based ACI.

These interventions might make CIK cell-based ACI an effective and routine therapy for cancer. In conclusion, CIK cell-based ACI has significant potential to improve clinical tumor therapy and to ameliorate the quality of life in patients with various malignancies.
